# Trench Foot Prevention: A Problem for Line Officers

**Published:** 1918-12

**Authors:** 


### TRENCH FOOT PREVENTION: A PROBLEM FOR LINE OFFICERS

That "trench foot" prevention is a problem for the line and quartermaster officers, rather than for medical men, was the outstanding feature of the symposium on that subject at the October meeting of the Research Society. It was brought out in the discussion that if soldiers are forced to bathe and dry their feet thoroughly, and to put on clean, dry socks, before going into the wet trenches, and to do likewise upon their return, provided that their feet are not wet and cold for too long a time, "trench foot" will not occur among soldiers. Representatives from the French and British Armies stated that during the first winter of the war trench foot was a considerable problem to both armies, but that when it was no longer considered merely a medical question, but the duty of line officers to see to its prevention, it became of rare concurrence at all times during the last three winters.

Medecin-Major Cottet of the French Army discussed the

" Etiology and Symptoms of Trench Foot ". He designated " trench foot " as a complex syndrome of vasomotor, nervous, and trophic disturbances, due to cold and dampness. He described the symptoms of the mild and severe types; and pointed out the many similarities between " trench foot " and Reynaud's disease.

Colonel Fuhr of the British Army was on the program for a paper on the " Prevention of Trench Foot ", but since he could not attend the meeting, his paper was read by Colonel Cummins. He brought out the fact that in the first winter of the war there was a great deal of trench foot among the British soldiers, but said that now it is a comparatively rare condition, and that, as soon as they leave the wet trenches, the soldiers are required to report for " foot-washing parade ". The company and platoon commanders supervise this ceremony, and see to it that the men wash and dry their feet thoroughly, after which they are provided with dry socks. When men are serving in the trenches, they are required to wash their feet and change socks and shoes or boots once or twice every day.

### Treatment of Trench Foot.

Colonel Bailey K. Ashford, Commanding Officer of the Field Sanitary School of the A. E. F., discussed " The Treatment of Trench Foot ", and in the course of his remarks said that this was largely symptomatic. He insisted that strict asepsis and inoculation with anti-tetanic serum are most important in dealing with " trench foot ". He believes that germs have nothing to do with its etiology, but that since the skin and subcutaneous tissues of the " trench foot " are in a state of lowered vitality, they frequently become infected with the staphylococcus, streptococcus and tetanus bacilli, and that the inflammatory reaction due to infection is often the most serious aspect of the condition.

Colonel Cummins said that the essential thing in the prevention of " trench foot " is to keep a layer of air rather than a layer of water next to the skin; and that so long as the skin has a layer of air next to it, it will bear conditions of cold very well, because it heats up the layer of air; but, when once water comes into contact with the skin, it loses its heat and lowered vitality ensues. He also called attention to the proper use of the long gum boot, which he said should be used only by men who are standing in the trenches. He mentioned an instance in the British Army when soldiers had marched for a considerable distance in the gum boots, their feet having gotten wet from sweat and then remained cold for some time, " trench foot " had resulted without any water having penetrated the boots. Colonel Cummins also spoke of the importance of thoroughly drying the long gum boot, and of issuing dry socks to the men before they go into the trenches, and immediately after they come out from them.

Sir David Bruce mentioned the fact that, during the first year of the war, quite a number of cases of tetanus had occurred among British soldiers who had " trench foot "; but that since the British Army has required that prophylactic doses of anti-tetanic serum be given soldiers who show symptoms of " trench foot ", no other cases of tetanus have occurred in patients suffering from that condition.

The papers and discussions on " trench foot " presented at the October meeting of the Research Society, and published in this number of *War Medicine*, give to American medical officers, and line and quatermaster officers, who should have an opportunity for reading them, the most important phase of a topic that is timely, even though the soldiers will not have to do any more fighting in trenches. The care of soldiers' feet in winter is important even under the most favorable conditions, and if the principles laid down by the medical authorities, who took part in the trench foot discussion, are followed by those who are responsible for the welfare of troops, their comfort will be considerably increased, and there will be a decrease in the present effective rates from foot diseases.

If medical officers desire copies of this number for transmission to their associated officers, copies will be furnished them. We should profit by the experience of our Allies in preventing unnecessary disablement of men from " trench foot ", a condition which is sure to occur even though fighting has ceased, unless the line and quartermaster officers co-operate in its prevention.

